# COVID-19 in India: Are Biological and Environmental Factors Helping to Stem the Incidence and Severity?

**DOI:** 10.14336/AD.2020.0402

**Published:** 2020-04-02

**Authors:** Sankha Shubhra Chakrabarti, Upinder Kaur, Anindita Banerjee, Upasana Ganguly, Tuhina Banerjee, Sarama Saha, Gaurav Parashar, Suvarna Prasad, Suddhachitta Chakrabarti, Amit Mittal, Bimal Kumar Agrawal, Ravindra Kumar Rawal, Robert Chunhua Zhao, Indrajeet Singh Gambhir, Rahul Khanna, Ashok K Shetty, Kunlin Jin, Sasanka Chakrabarti

**Affiliations:** ^1^Department of Geriatric Medicine, Institute of Medical Sciences, Banaras Hindu University, Varanasi, UP, India.; ^2^Department of Pharmacology, All India Institute of Medical Sciences, Gorakhpur, UP, India.; ^3^Department of Biochemistry, Institute of Post Graduate Medical Education and Research, Kolkata, West Bengal, India.; ^4^Department of Microbiology, Institute of Medical Sciences, Banaras Hindu University, UP, India.; ^5^Department of Biochemistry, All India Institute of Medical Sciences, Rishikesh, Uttarakhand, India.; ^6^Department of Biotechnology, Maharishi Markandeshwar (deemed to be) University, Mullana, Haryana, India.; ^7^Department of Biochemistry, MM Institute of Medical Sciences & Research, Maharishi Markandeshwar (deemed to be) University, Mullana, Haryana, India.; ^8^Health Department, Kolkata Municipal Corporation, West Bengal, India.; ^9^Department of Radiodiagnosis, MM Institute of Medical Sciences & Research, Maharishi Markandeshwar (deemed to be) University, Mullana, Haryana, India.; ^10^Department of General Medicine, MM Institute of Medical Sciences & Research, Maharishi Markandeshwar (deemed to be) University, Mullana, Haryana, India.; ^11^Department of Chemistry, Maharishi Markandeshwar (deemed to be) University, Mullana, Haryana, India.; ^12^School of Life Sciences, Shanghai University, Shanghai, China.; ^13^Department of Geriatric Medicine, Institute of Medical Sciences, Banaras Hindu University, Varanasi, UP, India.; ^14^Department of General Surgery, Institute of Medical Sciences, Banaras Hindu University, Varanasi, UP, India.; ^15^Institute for Regenerative Medicine, Department of Molecular and Cellular Medicine, Texas A&M University College of Medicine, College Station, Texas, USA.; ^16^Department of Pharmacology and Neuroscience, University of North Texas Health Science Center, Fort Worth, Texas, USA.; ^17^Department of Biochemistry and Central Research Cell, MM Institute of Medical Sciences & Research, Maharishi Markandeshwar (deemed to be) University, Mullana, Haryana, India.

**Keywords:** COVID-19, India, transmission, mortality, ACE2, cross-immunity

## Abstract

The ongoing Corona virus (COVID-19) pandemic has witnessed global political responses of unimaginable proportions. Many nations have implemented lockdowns that involve mandating citizens not to leave their residences for non-essential work. The Indian government has taken appropriate and commendable steps to curtail the community spread of COVID-19. While this may be extremely beneficial, this perspective discusses the other reasons why COVID-19 may have a lesser impact on India. We analyze the current pattern of SARS-CoV-2 transmission, testing, and mortality in India with an emphasis on the importance of mortality as a marker of the clinical relevance of COVID-19 disease. We also analyze the environmental and biological factors which may lessen the impact of COVID-19 in India. The importance of cross-immunity, innate immune responses, ACE polymorphism, and viral genetic mutations are discussed.

COVID-19 is an acute viral illness, instigated by a new coronavirus called the SARS-CoV-2 (severe acute respiratory syndrome coronavirus 2). The symptoms of COVID-19 range from mild to severe, and include mainly fever, cough, and respiratory distress. Severe cases with pneumonia and hypoxemia result in considerable mortality. The highest rate of infection has been seen in adults and aging individuals, but newborns and children could also be infected by SARS-CoV-2. Older individuals with pre-existing chronic health condition shave a higher risk of developing severe complications after SARS-CoV-2 infection. COVID-19 has emerged as a pandemic of respiratory illness ever since the first cases appeared in Wuhan, China, in December 2019 [1]. Currently, it has spread to 203 countries/territories worldwide, with >920,000 infected cases and >46,000 casualties (www.worldometers.info/coronavirus/).

This ongoing pandemic has witnessed global political responses of unimaginable proportions. Many nations have implemented lockdowns that involve mandating citizens not to leave their residences for non-essential work. The rules of lockdown vary from country to country. The Indian Prime Minister announced a nation-wide lockdown from the midnight of 24^th^ March 2020. Only essential services would be functioning and government services apart from health, law and order, banking, power and a few others have been suspended altogether. While the response by the Indian government both at the central and state levels has been commendable and timely given the global COVID-19 crisis, we deliberate some of the other reasons why the authors believe COVID-19 may have a lesser impact on India.

## 1. Low incidence of COVID-19 in India

**Figure 1.**
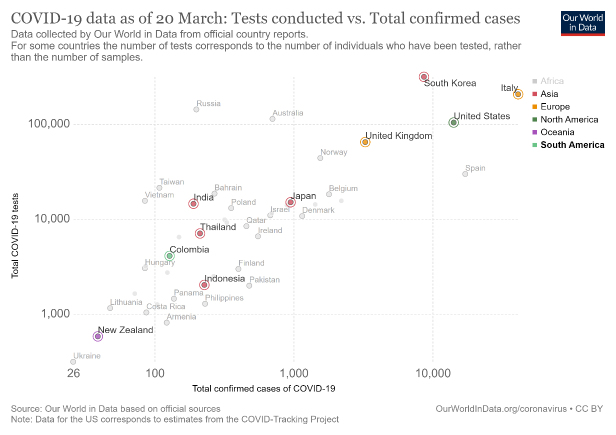
Representation of total COVID-19 tests performed in different countries for total confirmed cases of COVID-19. The status of India is shown with respect to other major nations. X-axis: total COVID-19 cases (logarithmic scale); Y-axis: total COVID-19 tests performed (logarithmic scale) (Figure courtesy https://ourworldindata.org/search?q=corona).

The world’s second-most populous country lies around the 40^th^ rank among nations for the number of diagnosed COVID-19 cases (www.worldometers.info/coronavirus/). The first case of COVID-19 was detected in India in the southern state of Kerala on 30^th^ January 2020 and was a medical student who had returned from Wuhan. Two other cases, also medical students back from Wuhan soon surfaced from Kerala (https://weather.com/en-IN/india/news/news/2020-02-14-kerala-defeats-coronavirus-indias-three-covid-19-patients-successfully). The Indian government implemented thermal screening of inbound international air-travelers. Such measures were implemented for China around17^th^January and then, in a stepwise fashion, expanded to include all international passengers (https://pib.gov.in/PressReleaseIframePage.aspx?PRID=1599901). Interestingly, thermal screening is far from a fool-proof way to detect SARS-CoV-2 carriers, many of whom may be in the middle of the incubation period or mild cases and hence asymptomatic. Further, the quarantine of international travelers on arrival was initiated only for those from China and its immediate neighbors at the beginning and then expanded for travelers from other countries, that too in a phased manner, while observing global trends of COVID-19 spread. Domestic travel in India continued unabated without incorporating any screening. Further, the initial testing criteria with confirmatory qRT-PCR (quantitative reverse transcription polymerase chain reaction)-based tests for suspected patients, released by the Indian Council of Medical Research (ICMR), emphasized testing only those with laboratory-proven contacts or recent foreign travel (www.icmr.nic.in/content/covid-19). With such restrictions, the movement of several SARS-CoV-2 positive patients, whether asymptomatic or symptomatic, must have occurred all over India. However, 9 weeks after the first case was diagnosed, India has a COVID-19 count of 2300 (www.mohfw.gov.in/). In contrast, the first two Italian cases were Chinese tourists who arrived in Milan on 23^rd^ January, and tested positive on 30^th^ January, the same time when the first Indian case was recorded [2]. The case positivity in Italy exploded after the fourth week since March 1^st^ when daily more than 250 new cases (more than 500 new cases daily since 4^th^ March) were diagnosed (https://ourworldindata.org/coronavirus). It is curious that among the two countries which reported their first case at almost the same time, the progression has been so disparate. One may argue that testing in India has been sluggish in contrast to aggressive testing by some developed nations. However, India had tested samples (nasal/throat swabs for qRT-PCR) from 26798 patients suspected to have COVID-19, as of 27^th^ March which still gave a testing positivity rate of 2.6%, a figure which has not increased significantly over time or with changes in testing criteria (https://icmr.nic.in/sites/default/files/whats_new/ICMR_website_update_27March_9AM_IST.pdf). A further update by the ICMR pegged the figure at 42788 samples by 30^th^ March (2.5% test positivity). In comparison, Italy at the top of the spectrum had carried out nearly 206886 tests as of 20^th^ March with 41035 positives, which translates into a positivity rate of tested individuals of 19.8%. South Korea, on the other hand, adopted a comprehensive strategy of community testing. Such an approach translated into 8652 positives in 316664 tests (2.7%) (www.statista.com/statistics/1028731/covid19-tests-select-countries-worldwide/). A few other countries at different ends of the spectrum are represented in the graph in Figure 1. One instantly notices the surprisingly low rates of COVID-19 positivity in India, and the South Korean example implies that higher testing may not necessarily alter the incidence rates of COVID-19. This low rate of COVID-19 positivity remaining constant over a couple of weeks has led most experts to believe that community spread of the disease has not taken place in India as yet. Such inference is, however, intriguing and needs careful analysis. The reports of hospitalization of COVID-19 patients appeared in China from mid-December 2019 and given the average incubation period of 14 days for the manifestation of the disease, the viral spread likely started in China in early December 2019 [1]. The geographical proximity of India to China and the regular unrestricted movement of people between two countries through multiple daily direct and indirect flights throughout December 2019 until mid-January 2020 should have made a densely populated nation like India rapidly invaded by COVID-19. Considering these, we propose several possibilities for the low incidence of COVID-19 in India, which include both environmental and biological factors.

## 2.1. Environmental and Demographic factors

The population density of India is 414 persons/km^2^, which is higher than the six countries with maximum COVID-19 cases. Such a finding is a paradox as countries with higher densities and people staying closer to each other are theoretically at a higher risk of contracting communicable diseases transmitted by fomites or aerosols. Similarly, the examples of South Korea (517 persons/km^2^) and Japan (333 persons/km^2^), which have a relatively lower incidence of COVID-19 and a higher population density, support this curious case. Trying to explain this paradox would require further research.

In the Köppen classification of climatic zones of the world, both Wuhan, the apparent epicenter of the pandemic and most of Italy are categorized as type C (mild temperate) whereas much of India has either a dry (type B) or tropical climate (type A). However, this again raises queries on why Iran having a dry desert climate (type B) bore the brunt of COVID-19 cases [3]. It has been suggested that high temperatures and high relative humidity levels significantly reduce SARS-CoV-2 transmission [4]. It may be fortuitous for India that as it enters the weeks deemed to mark its entry into phase 3 of the epidemic, the climate over large portions of the country is taking a turn for this same high temperature, high humidity state.

## 2.2. Biological factors

### 2.2.1. Biology of SARS-CoV-2 and cross-immunity

Like other animal and human corona viruses, SARS-CoV-2 is also a positive-strand RNA (approximately 30kilobases) virus which codes for a replicase enzyme associated with other enzymatic activities and several structural proteins of which the spike protein (S) is essential for viral entry into host cells [5]. SARS-CoV, HCoV-NL63 and SARS-CoV-2 enter the host cells through the protein angiotensin 1 converting enzyme 2 (ACE2) on the cell membrane and the viral entry requires the cleavage of S protein by a serine protease (TMPRSS2) [5,6]. ACE2 protein is expressed in many different types of cells, and the virus can invade multiple organs [1]. Since pulmonary alveolar cells and mucosa of the gastro-intestinal tract have high expressions of ACE2, SARS-CoV-2 can easily invade these organs [1, 7]. The invasion of lungs leads to the most critical pathology of pneumonia with severe respiratory distress and hypoxemia that accounts for the significant mortality in this disorder. Enhanced production of pro-inflammatory cytokines and chemokines by immune effector cells leading to a cytokine storm is thought to be the cause of multiple systemic and respiratory symptoms. The genomic sequences of SARS-CoV-2 from multiple isolates show more than 99% identity which is sufficiently different from other human corona viruses and its origin has been traced to a bat corona virus [1]. Apart from SARS-CoV and MERS-CoV which caused severe respiratory diseases following outbreaks in 2003 and 2012, there are four endemic human corona viruses, HCoV-229E, HCoV NL-63, HCoV-OC4, HCoV-HKU1 in populations that are responsible for various types of respiratory illness which are generally self-limiting in young and immune-competent persons [8].

The defense against any viral infection involves both innate immunity and adaptive immunity. While adaptive immunity relies on antibodies and T-cells which can recognize viral antigens with high degrees of specificity, the innate immunity utilizes receptors (Pattern Recognition Receptors of several types) which recognize broad structural motifs present in bacteria or viruses but generally absent in host cells. There are currently no established data on the specific role of either humoral or cellular immunity or innate immunity in patients recovering from COVID-19, but immune response associated with SARS-CoV and MERS-CoV has been studied in some detail [9]. It can be assumed that some degrees of sequence homology or conformational similarities among the structural proteins, especially the S protein, of SARS-CoV-2 and the endemic corona viruses (HCoV-229E, HCoV NL-63, HCoV-OC4, HCoV-HKU1) may result in cross-reactive immunity (circulating antibodies or primed T-cells) in persons with prior exposure to the latter viruses, and this may modulate the course and outcome of COVID-19. This kind of cross-reactive immunity modulating the host-response to viral infection is well-known and widely studied in infections with flaviviruses (between different subtypes of Dengue viruses or between the Dengue virus and Zika virus) [10,11]. Although the cross-reactive immunity may be protective in some cases, it may lead to augmented harmful reactions in other cases which has been termed antibody-dependent enhancement (ADE), and these processes have been established from epidemiological studies as well as in animal models [10,12]. Thus, the possibility of a protective cross-immunity in the Indian population against COVID-19 cannot be ignored in explaining a rather mild effect of the current coronavirus pandemic in India in comparison to that in Europe and the USA. It is important to note that the presence of cross-reactive antibodies in the sera of patients infected with different types of human corona viruses have not been studied extensively. However, one study reported that sera of subjects who had SARS-CoV infection developed cross-reactive antibodies to HCoV-229E and HCoV-OC43 [13]. Similarly, sera of convalescent patients of SARS-CoV have been shown to contain neutralizing antibodies against MERS-CoV [14]. Likewise, sera of convalescent patients of SARS-CoV contain neutralizing antibodies that inhibit the entry of SARS-CoV-2 in Vero cells [6]. Therefore, cross-reactive antibodies generated as a result of infections from other human corona viruses may have a protective role in a population affected by COVID-19. Of note, antibodies against MERS-CoV have been detected in a significant fraction of persons exposed to camels and dromedaries without any clinical evidence of prior MERS suggesting that MERS-CoV can infect individuals without any symptoms, and yet induce signs of protective immune response [15].

It is noteworthy that many corona viruses are widely present in cattle, pigs and chickens producing a variety of diseases affecting the respiratory and gastro-intestinal systems in these species [5]. The animal corona viruses do not infect human beings unless a mutation breaks the species-barrier. Nonetheless, a large number of people working in dairy farms and livestock sectors come in close contact with animals and extensively handle the raw meat of these species in markets and houses with bare hands, which may expose the circulating immune cells to viral proteins through minor injuries in the skin. Such exposures may result in the development of cross-immunity because of possible antigenic similarities among human and animal corona viruses.

Another interesting observation is the apparent exclusion of malaria-endemic zones by the COVID-19 epidemic. India, large parts of Africa, and parts of South America that report ongoing malaria transmission have had a low incidence of COVID-19. The reasons are not known, but an explanation may lie in the ensuing cytokine storm, that may be involved in the severe respiratory manifestations of COVID-19 [16]. It is now widely accepted that not only malaria but many other acute infective conditions such as sepsis have deleterious effects mediated through the human body’s response to the infective agent, in the form of a cytokine storm [17]. It is plausible that persons in malaria-endemic zones, which may also be endemic for several tropical pathogens including viruses, have recurrent cytokine fluctuations in response to minor and subclinical infections. These recurrent fluctuations may have a de-sensitizing effect on the body’s immune system, which prevents an uncontrolled and detrimental immune response and thus severe clinical disease in SARS-CoV-2 infection. Hence, many infected patients, may not manifest any symptoms and, as a corollary, may not meet testing criteria, resulting in a low positivity rate. It may be mentioned here that a novel therapeutic approach has been tried recently in COVID-19 utilizing the immunomodulatory role of mesenchymal stem cells to attenuate the cytokine storm [18].

### 2.2.2. ACE2 polymorphism

Apart from cross-immunity, an essential mechanism of the variable outcome of COVID-19 may lie in the polymorphism of ACE2 protein, which is coded by a gene on the X-chromosome. Several single nucleotide polymorphisms of this protein have been described which may have implications in altered expression levels of ACE2 or its interaction with S protein of SARS-CoV-2. However, a detailed analysis of such polymorphisms in different populations *vis-à-vis* the outcome of COVID-19 will require more time [19]. Further, in viral infections, host antiviral miRNAs play a crucial role in the regulation of immune response, depending upon the viral agent. Many known human miRNAs appear to be able to target viral genes and their functions by interfering with replication, translation, and expression. In this context, an interesting, but yet to be peer-reviewed research article, performing a sequence-based analysis has reported a unique mutation in the Indian SARS-CoV-2 strain affecting the S protein of the virus that allows it to be a target for a miRNA (hsa-miR-27b) [20].

### 2.2.3. Possibility of missing COVID-19 with currently employed tests

While not directly relevant to issues discussed in this article, a possibility also exists that many cases may be missed due to the sensitivity of the tests being performed. Mostly pharyngeal and nasal swabs are used for qRT-PCR based diagnostics. A recent article mentions these samples as having only 32% and 63% positivity while testing for COVID-19 [21].

**Table 1:** Demographic characteristics, travel history and health status of patients dying with COVID-19 positivity in India.

Age (years)	Gender	State	History of foreign travel in past 3 weeks	Co-morbidities
38	M	Bihar	Yes, returned from Qatar	Renal disease
68	F	Delhi	Son returned from Switzerland	Diabetes mellitus, hypertension
70	M	Gujarat	No, but domestic travel to Delhi^#^	Cancer, diabetes mellitus, hypertension
69	M		No, but domestic travel to Delhi & Jaipur^##^	Asthma, kidney disease, sepsis, septic shock
85	F		Yes (details NM)	Yes, details NM
45	M		NM	Diabetes mellitus
46	M		NM	Diabetes mellitus, hypertension, pulmonary fibrosis
69	M	Himachal Pradesh	Yes, returned from US	------
65	M	Jammu & Kashmir	No, but domestic travel to multiple cities including Delhi	---- Died of cardiac arrest
50s-60s, confusion in age	M		No, met a couple from Saudi Arabia	Liver disease
76	M	Karnataka	Yes, returned from Saudi Arabia	Asthma
70 or 75	F		Yes, returned from Mecca, Saudi Arabia	Diabetes mellitus, chest pain, hip fracture
65	M		No, but domestic travel to Delhi	NM
69	M	Kerala	Yes, returned from Dubai	Hypertension, brought dead to hospital
35 or 65, (confusion in age)	M	Madhya Pradesh	No	Yes, NM
65	F		No	---
47	M (suspected)		No	---
65	F	Maharashtra	No	----
65	F		No	----
68	M		Yes, returned from Philippines	Diabetes mellitus, asthma, acute renal failure
63 or 64 (confusion in age)	M		Yes, returned from Dubai	Diabetes mellitus, hypertension, ischaemic heart disease
40	F		NM	Hypertension, chest pain for last few days
71	M		Yes, returned from Saudi Arabia	Diabetes mellitus, hypertension, CABG
45^**^	M		NM	NM, yet to be found
70 or 72 (confusion in age)	M	Punjab	Yes, returned from Germany & Italy	---- Died of cardiac arrest
73	M	Rajasthan	NM	Diabetes mellitus, renal failure
54	M	Tamil Nadu	NM	Steroid dependant COPD, diabetes mellitus, hypertension
74	M	Telangana	No, but domestic travel to Delhi	Yes, NM, Brought dead to hospital
57	M	West Bengal	No, but domestic travel to Bilaspur^*^	---- Died of cardiac cause

(N=29, Last update as of 29^th^ March 2020) Data is procured from official website for COVID-19 in India (www.Covid19india.org) and from news reports. Data may not be fully accurate as official data has not been released yet. CABG, coronary artery bypass graft; COPD, chronic obstructive pulmonary disease; F, female; M, male; NM, Not mentioned

# National capital of India

## Place in Rajasthan State of India

* Place in Uttar Pradesh State of India

** Not updated as of 29 March 2020

### 2.3. COVID-19 mortality- why the Indian cloud seems to have a silver lining

Another vital aspect of COVID-19 is mortality in diagnosed cases. Mortality is the statistic that is most relevant for developing nations such as India. Acute viral illnesses as well as acute infections due to any pathogenic agent, are prevalent in India, owing to dismal standards of hygiene, especially among a large section of its population which lives below the poverty line. An exact number cannot be ascribed to the incidence of viral infections, because of the sheer diversity of such infections ranging from respiratory viral infections (influenza, rhinovirus, adenovirus, coronavirus, etc.) to several arthropod-borne viral fever syndromes. The diagnostic facilities are non-existent in the periphery, and the majority of these infections may be subclinical or may present with atypical manifestations [22]. An idea can be obtained from the data of the Integrated Disease Surveillance Programme (IDSP) of the Ministry of Health and Family Welfare (MoHFW) of the government of India. During 2017, the IDSP reported 1683 disease outbreaks in India, of which 71% were due to viral pathogens [23]. The authors, in their clinical practice, also treat patients regularly who present with classic symptoms of viral respiratory disease. Sometimes, the same patient with comorbidities such as COPD visiting multiple times in a year. Whereas such patients in affluent nations would be subjected to additional testing and probably annual influenza vaccination, such measures are mostly non-existent in the Indian scenario, apart from a few of the apex centers. The majority of such patients with fever, upper and lower respiratory tract symptoms, bodyache and headache may either not turn up at clinics, dismissing their symptoms as another bout of viral fever and resorting to home remedies, or even in case they present to a trained practitioner, supportive management is at best done. Even drugs such as oseltamivir are scarcely used in routine practice. Even with community transmission of the virus (phase 3 of the epidemic), most cases may turn out to be subclinical and mild. In the end, mortality and not morbidity is the statistic that matters. Such a scenario brings us to the critical question; what is the actual attributable mortality of COVID-19? A peek into the Italian scenario may be helpful. Nearly 85% of COVID-19-related deaths in Italy have been individuals in the 70+ years age group. Among the deceased patients, chart details were available for 481. Out of these only 6 patients did not have any comorbidities. Among the 9 patients of age < 40 years who died (M=8, F=1), clinical information was available for 7, and each had pre-existing serious comorbidity(www.epicentro.iss.it/coronavirus/bollettino/Report-COVID-2019_20_marzo_eng.pdf).

Even in India, more than half of the deaths that have ensued have affected patients more than 60 years of age, the accepted geriatric age cutoff in the country. Although the chart details of the Indian patients are unavailable, the available data collected from reputed news sources is presented in Table 1. Most cases with fatality, whether young or old evidently had comorbidities. It is common sense that in a patient with a fatality, in the presence of severe renal or pulmonary or cardiac disease, the mere presence of COVID-19 positivity does not confirm the role of coronavirus in causing death. Detailed case histories may be revealed in the coming days, which may further clarify the matters.

**Figure 2.**
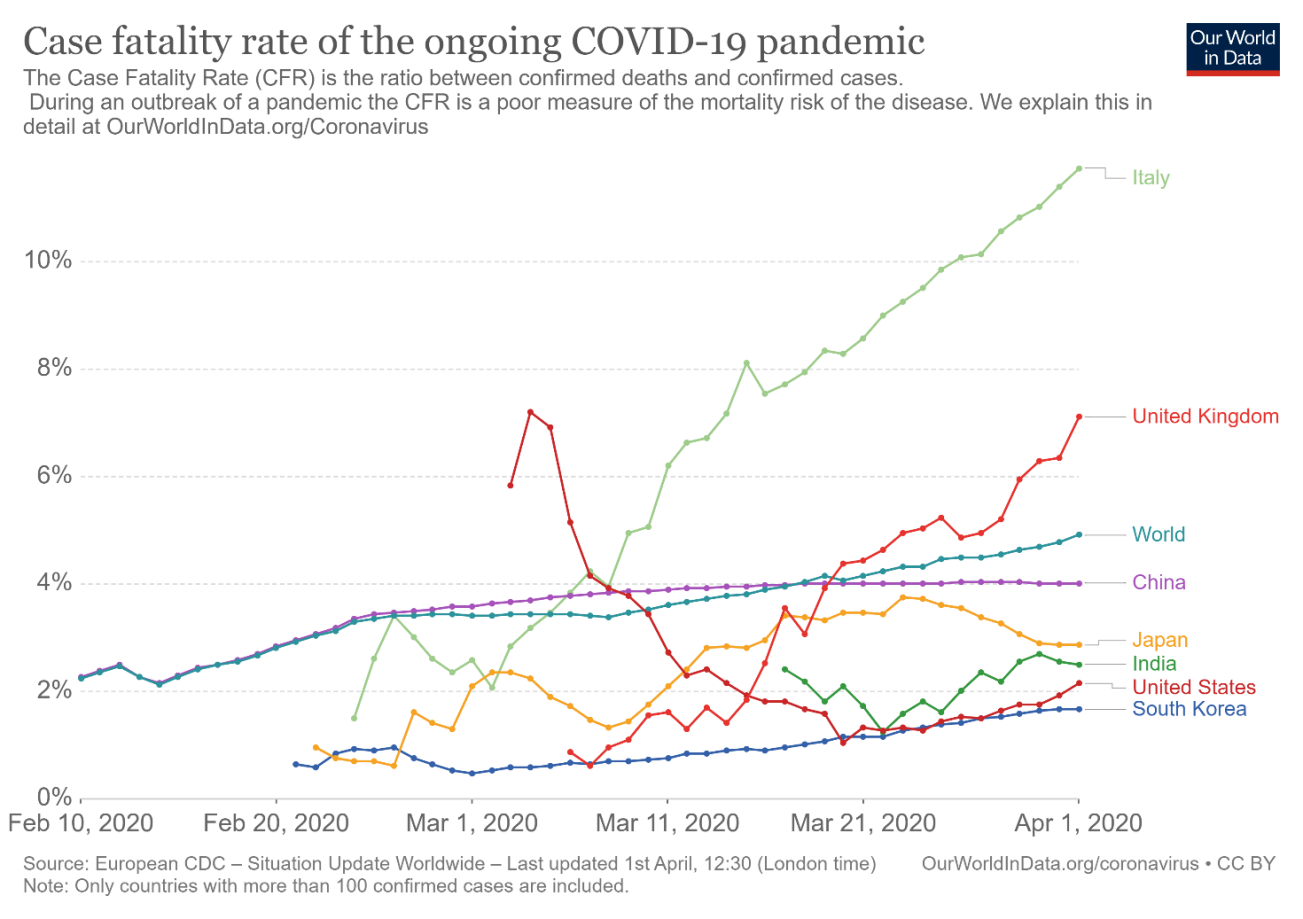
Representation of country-wise timeline of COVID-19 case fatality rate. The status of India is shown with respect to other major nations. (Figure courtesy https://ourworldindata.org/search?q=corona).

**Figure 3.**
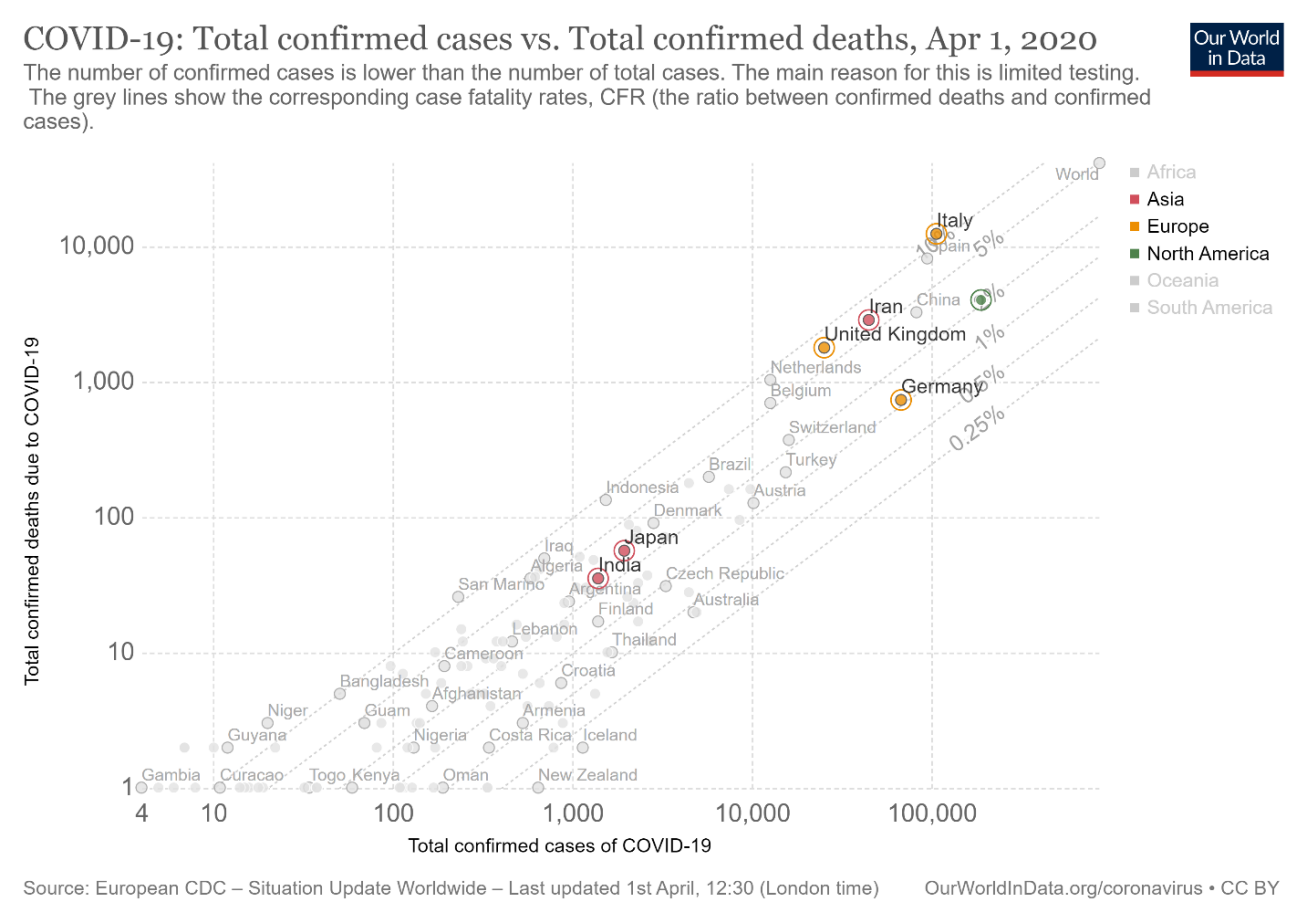
Representation of total confirmed cases with respect to total confirmed deaths in India, in comparison with other major nations. X-axis: total COVID-19 cases (logarithmic scale); Y-axis: total confirmed deaths due to COVID-19 (logarithmic scale) (Figure courtesy https://ourworldindata.org/search?q=corona).

There may be a faulty statistical angle to COVID-19 mortality in India too. Italy (23%) stands second in the world after Japan (27%) in having a population of > 65 years age (www.worldatlas.com/articles/countries-with-the-largest-aging-population-in-the-world.html). In life-expectancy it also ranks second after Japan among all countries with a population more than 10 million. Italian healthcare provides for universal coverage and is largely free. What this means is a sizeable section of the population in Italy has survived to an age that would be impossible for them to do up to, in other countries. Contrast this to the situation in India where healthcare services do not have this much peripheral penetration. Further, transport and awareness issues as well as societal preferences for the younger earning population, results in neglect of the elderly and failure of free healthcare to translate into health gains for the older population. India’s life expectancy stands at 69 years, interestingly less than the mean age of COVID-19 fatalities in Italy. So, India may not have the most vulnerable age group for COVID-19 to exert its effect to the fullest. Overall, if one analyzes the mortality statistics of India in comparison to other countries (Fig. 2), it is clear that the curve has been flat for quite long. A plot between total confirmed cases and total confirmed deaths clarifies the situation in India further in comparison to other major countries (Fig. 3).

## 3. Conclusion

The different restrictive measures adopted by the central and the state governments in India like visa and travel restrictions, home isolation and quarantine, and finally nation-wide lockdown are classical methods of prevention of the community spread of SARS-CoV-2. These methods must have played a role in limiting the effect of this pandemic on the Indian population. However, we strongly feel that various biological and environmental factors have also contributed significantly to this process, which may result in COVID-19 in India behaving more mildly in contrast to its global effects. The careful analysis of such biological factors will certainly open new avenues to combat outbreaks of novel viruses, through research on cross-immunity, immuno-modulation and perhaps life-style management and dietary manipulations.
